# HEK293T Cells Are Heterozygous for CCR5 Delta 32 Mutation

**DOI:** 10.1371/journal.pone.0152975

**Published:** 2016-04-04

**Authors:** Chunxia Qi, Xiaopeng Jia, Lingling Lu, Ping Ma, Min Wei

**Affiliations:** 1 School of Medicine, Nankai University, Tianjin, China; 2 Department of Infectious Disease, The Tianjin Second People’s Hospital, Tianjin, China; Temple University School of Medicine, UNITED STATES

## Abstract

C-C chemokine receptor 5 (CCR5) is a receptor for chemokines and a co-receptor for HIV-1 entry into the target CD4+ cells. CCR5 delta 32 deletion is a loss-of-function mutation, resistant to HIV-1 infection. We tried to induce the CCR5 delta 32 mutation harnessing the genome editing technique, CRISPR-Cas9 (Clustered Regularly Interspaced Short Palindromic Repeats, CRISPR and CRISPR associated protein 9, Cas9) in the commonly used cell line human embryonic kidney HEK 293T cells. Surprisingly, we found that HEK293T cells are heterozygous for CCR5 delta 32 mutation, in contrast to the wild type CCR5 cells, human acute T cell leukemia cell line Jurkat and human breast adenocarcinoma cell line MDA-MB-231 cells. This finding indicates that at least one human cell line is heterozygous for the CCR5 delta 32 mutation. We also found that in PCR amplification, wild type CCR5 DNA and mutant delta 32 DNA can form mismatched heteroduplex and move slowly in gel electrophoresis.

## Introduction

Human Immunodeficiency Virus (HIV) infection and Acquired Immunodeficiency Syndrome (AIDS) is characterized by progressive CD4+ T cell depletion in patients’ peripheral blood and lymphoid tissues. [1–5]. The pathogen of AIDS is HIV [1]. HIV entry into CD4+ cells is dependent on the receptor CD4 and co-receptors, such as CCR5 (C-C chemokine receptor 5) or CXCR4 (C-X-C chemokine receptor type 4) on the surface of target cells [4, 6–9]. CCR5-tropic viruses predominate most isolates from human bodies globally and usually found in the early HIV infection. CXCR4-tropic viruses are fewer, and usually found in the late stage of infection [4, 9, 10]. CCR5 delta 32 mutation is a deletion of 32 base pairs after amino acid position 185, resulting in a premature and non-functional CCR5 receptor [9, 11]. Homozygous CCR5 delta 32 (CCR5 delta32/delta32) individual was resistant to CCR5-tropic HIV infection, and heterozygous carriers were slow in AIDS progression [4, 11–13].

In 2009, German doctors reported that a Berlin patient, who suffered chronic HIV infection and acute myeloid leukemia, got a bone marrow transplant. The donor was homozygous for CCR5 delta 32 mutation [14]. After the transplantation, the Berlin patient is still healthy [14]. Most importantly, he is still healthy after 7 years of stopping the antiretroviral therapy [15]. Now, he is considered the first and only cured HIV-infected patient in the world [15–17]. The success of Berlin patient stimulates the scientists’ interest to disrupt CCR5 gene and even integrated HIV genome by up-to-date genome editing techniques, such as zinc finger nuclease (ZFNs), or transcription activator-like effector nuclease (TALENs), or RNA-guided CRISPR-Cas9 nuclease techniques [18–25].

We also tried to convert wild type CCR5 gene into mutant CCR5 delta 32 using CRISPR-Cas9 technique. However, we accidentally found that commonly used normal HEK293T cells are heterozygous for CCR5 delta32 mutation.

## Material and Methods

### Cell Culture

Human embryonic kidney HEK293T cells (ATCC, CRL-11268), human acute T cell leukemia cell line Jurkat clone E6-1 (ATCC, TIB-152), and human breast adenocarcinoma cell line MDA-MB-231 (ATCC, HTB26) were obtained from American Type Culture Collection (ATCC). The cells were maintained in DMEM (Gibco) or RPMI1640 (Gibco) media containing 10% fetal bovine serum (Gbico) and 1% Penicillin- Streptomycin (Gbico) according to the protocols (http://www.atcc.org).

### Construction of vectors, TA cloning and sequencing

First, genomic DNA was extracted from cells with the Multisource Genomic DNA Miniprep Kit (Axygen), and then amplified by PCR with forward primer 5^,^TCTTCTTCATCATCCTCCTG 3^,^ and reverse primer 5^,^ GTTTGGCAATGTGCTTTT 3^,^. The PCR products were resolved in 1.7% agarose gel. Target bands were cut and purified with DNA Gel Extraction Kit (Axygen), and cloned into TA vector (CloneJET PCR cloning Kit, Ferments). After TA vector transformation into DH5α *E*.*coli* and incubation at 37°C overnight, we picked and sequenced several bacterium colonies.

### T7 endonuclease I (T7E1) digestion Assay

Genomic DNA extraction and PCR amplification were described as above. PCR products were then purified with DNA Gel Extraction Kit (Axygen). The purified PCR products 400ng were mixed with 2μl 10× T7E1 nuclease buffer and nuclease-free water to a volume of 19μl. These products were denatured for 10 min at 95°C, annealed by gradual cooling in a thermocycler and digested by 1μl T7E1 nuclease (GeneCopoeia^TM^). The digestion was performed at 37°C water bath for 40 minutes, and followed by analyzing in 1.7% agarose gel.

## Results

### CCR5 gene mutation exists in HEK293T cells

**Fig 1:**
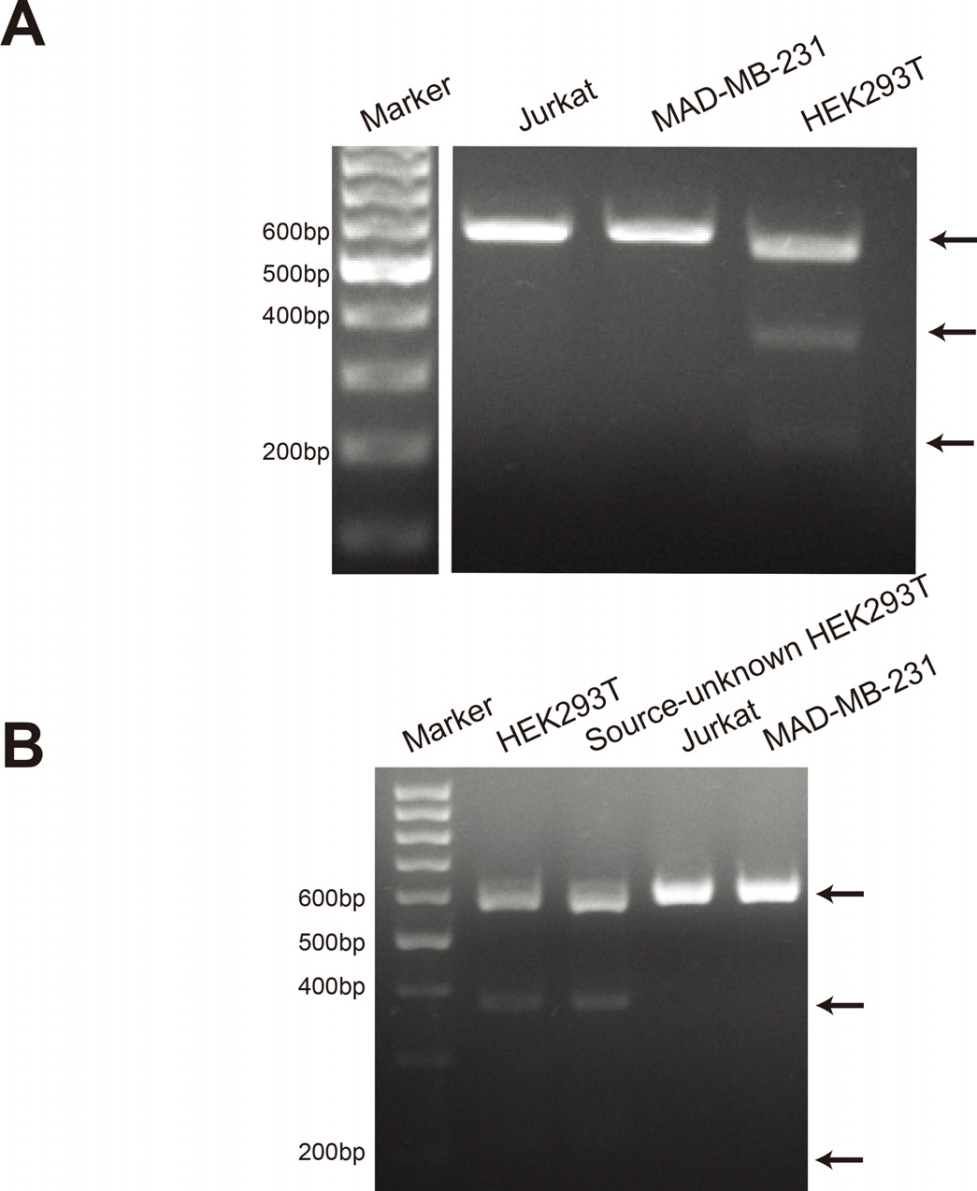
CCR5 gene mutation exists in HEK293T cells. (A) CCR5 gene from Jurkat, MAD-MB-231 and HEK293T cells was amplified by PCR. The PCR products were digested by T7E1 and analyzed in 1.7% agarose gel. The PCR product bands and digested bands are indicated by arrows. (B) The experiment was repeated with another source-unknown HEK293T cells.

We tried to convert CCR5 gene into homozygous CCR5 delta 32 mutation in HEK293T cells, using CRISPR-Cas9 technique. T7E1 digestion assay is usually used in CRISPR-Cas9-induced mutation detection. T7E1 can recognize heteroduplex DNA and cleave mismatched regions. When we amplified CCR5 gene from HEK293T cells, we accidently found that CCR5 PCR products from HEK293T cells can be digested by T7E1 (Fig 1A). This phenomenon only appeared in HEK293T cells, but not in Jurkat cells and MAD-MB-231 cells (Fig 1A). To further confirm this, we analyzed another source-unknown HEK293T cells obtained from another laboratory. The same results were acquired (Fig 1B). This suggests that CCR5 allele in HEK293T cells contains a mutation.

### HEK293T cells are heterozygous for CCR5 delta32 mutation

**Fig 2:**
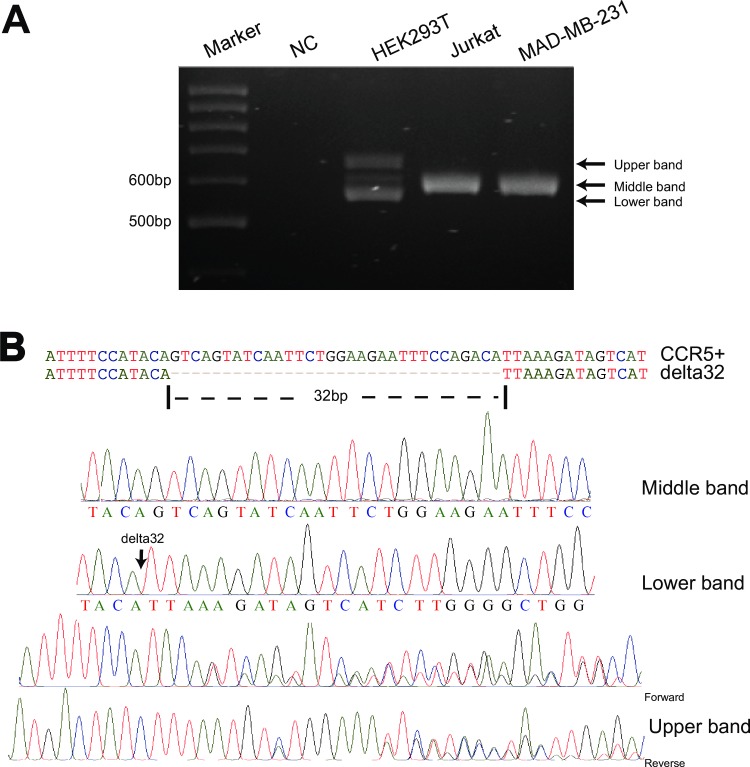
HEK293T cells are heterozygous for CCR5 delta32 mutation. (A) The CCR5 PCR products from Jurkat, MAD-MB-231 and HEK293T cells were resolved in 3% agarose gel. Upper, middle and lower bands are indicated. “NC” stands for no template negative control. (B) Sequencing chromatograms from the middle, lower, and upper bands (two directions) are showed.

To further confirm the CCR5 mutation in HEK293T cells, the above CCR5 PCR products were resolved in 3% agarose gel. Surprisingly, three different bands were observed in the results. The middle band was same as the band amplified from Jurkat cells (Fig 2A). Since there was one band below and one band above the wild type band respectively, we could not ascertain the type of mutations, insertion or deletion.

We then cut each band, purified, inserted into TA vectors, and transformed into DH5α *E*.*coli* for sequencing. As expected, the middle band was wild type CCR5 sequence, the lower band was CCR5 delta 32 deletion (Fig 2B). However, sequencing data from the upper band were complicated. In total fifteen single *E*.*coli* colonies, four colonies were wild type CCR5, six colonies were delta 32 deletion, and all the remaining five colonies gave noisy signals in sequencing chromatograms both from forward and reverse directions (Fig 2B), even though we picked a single colony for sequencing. When we read the noisy chromatogram carefully, we found that the noisy signal started from the position of delta 32 deletion, suggesting that the sequencing sample contained a mixture of samples.

### Identification of three bands produced from the results of DNA electrophoresis

**Fig 3:**
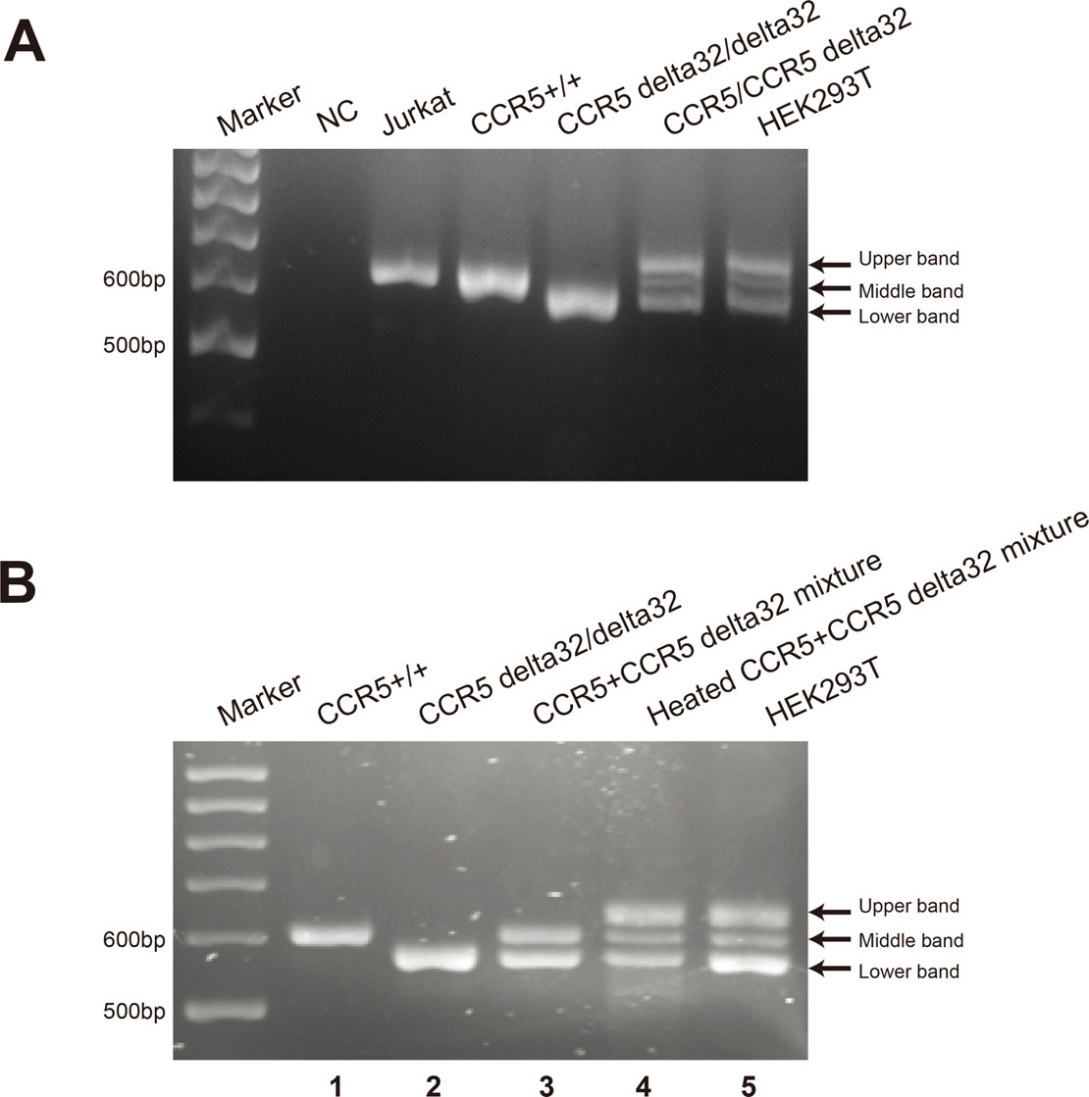
The upper band is heteroduplex of wild type and mutant CCR5 delta 32. (A) 3% agarose gel electrophoresis of PCR products are showed from Jurkat, HEK293T cells, wild type CCR5 plasmid (CCR5+/+), CCR5 delta 32 plasmid (CCR5 delta 32/delta 32), and the template mixture of wild type CCR5 and CCR5 delta 32 (CCR5/CCR5 delta 32). “NC” represents no template negative control. (B) 3% agarose gel electrophoresis of PCR products are indicated respectively from PCR amplification of wild type CCR5 plasmid (lane 1, CCR5 +/+), CCR5 delta 32 plasmid (lane 2, CCR5 delta 32/delta 32), and HEK293T cells (lane 5). Lane 3 shows the unheated PCR product mixture of wild type CCR5 and delta 32 (CCR5+CCR5 delta 32 mixture, samples from lane 1 and lane 2). Lane 4 shows the heated PCR product mixture of wild type CCR5 and delta 32 (Heated CCR5+CCR5 delta 32 mixture, samples from lane 1 and 2).

These data show that HEK293T cells are heterozygous for CCR5 delta 32. However, the third upper band still puzzled us. Theoretically, PCR products from HEK293T cell should produce two bands when analyzed in agarose gel, but actually three bands appeared. To answer this question, we mixed the plasmid containing wild type CCR5 sequence and the plasmid containing mutant CCR5 delta 32 in an equal amount. We used the mixture as a template in PCR amplification. Interestingly, this mixture showed the same result as HEK293T cells (Fig 3A). This data implies that HEK293T cells have equal template of wild type CCR5 and mutant CCR5 delta 32, which confirmed the conclusion of CCR5 delta 32 heterozygosity of HEK293T cells. Next, we directly mixed the PCR products from wild type and mutant CCR5 delta 32 in an equal amount. This mixture was divided equally. One remained at room temperature and the other was denatured at 95°C for ten minutes, cooled gradually, and then analyzed on 3% agarose gel. We found that only the heated PCR product mixture emerged the third band, but the mixture without heating did not show the third upper band (Fig 3B, compare lane 3 and 4). Again, we cut the third upper band, cloned into the vector, transformed into *E*.*coli*, incubated overnight and picked six single colonies for sequencing. The chromatograms from all colonies gave noisy signals again, the same as the third band from HEK293T cells. These data indicate that the third band is heteroduplex double strand DNA between the wild type and mutant CCR5 delta 32. The herteroduplex DNA always move more slowly than homoduplex DNA in electrophoresis.

## Discussion

To date, although antiretroviral therapy (ART) is employed to treat HIV/AIDS patients and control viremia effectively, ART can not completely eliminate integrated proviral DNA [2, 16]. Therefore, AIDS is still currently incurable. The Berlin patient success encourages scientists to cure AIDS by genome editing techniques [14, 25].

In this study, we accidentally found natural HEK293T cells are heterozygous for CCR5 delta 32 mutation, and this mutation does not exist in Jurkat cells and MDA-MB-231 cells. We detected three bands in PCR amplification of CCR5 allele from HEK293T cells, which was also found in Hutter’s study [14]. They speculated the third band was an artifact, but did not prove it. Here we provide the experimental evidence to prove the third band is the heteroduplex of wild type and mutant CCR5 delta 32. The third band could not be separated well if the agarose gel was less than 2% [26]. In Fig 3B, only the mixture of PCR products from wild type and mutant CCR5 treated at 95°C, produced the third band; but the untreated mixture did not. This data indicate that high temperature can denature the double strand DNA, and form the mismatched heteroduplex of wild type and mutant CCR5. It also proves that the third band is an artifact during the PCR amplification, because PCR also undergoes repeated cycles on denaturing step typically at 94–96°C, annealing step at 50–65°C, and extension step at 72°C.

Surprisingly, it is unexpected that DNA sequencing of the single bacterium colony containing CCR5 heteroduplex produces noisy signals. We even picked the single colony with noisy signals in sequencing, streaked inoculation in a new LB plate, grew overnight, picked single colony and sequenced again. In total twenty single colonies, one (5%) was wild type CCR5, one (5%) was delta 32 mutation, and the remaining eighteen (90%) colonies were heteroduplex, producing noisy signals in sequencing again (Table 1). In another repeated experiment, similar results were obtained, 10% (2/20) was wild type, 10% (2/20) was delta 32 mutation, and 80% (16/20) was heteroduplex (Table 1). Our explanation is that two types of plasmids wild type and mutant CCR5 could go into one bacterium, and replicate to form one single colony.

**Table 1:** Streak inoculation and single *E*.*coli* colony sequencing from a single colony with noisy signals in sequencing.

Test	CCR5 wild type	CCR5 delta 32 mutation	Heteroduplex of CCR5 WT/delta32	Total colonies
1	1 (5%)	1 (5%)	18 (90%)	20
2	2 (10%)	2 (10%)	16 (80%)	20

HEK293T cells could come from a heterozygous individual of CCR5 delta 32 mutation. The average frequency of CCR5 delta 32 mutation is around 10% in European populations, but it is almost absent in African, American Indian, and Asian populations [27, 28]. It is still not clear that the occurrence of CCR5 mutation is under which kind of nature pressures. Many hypotheses were raised to explain the higher mutation frequency in Europe. One is the selective pressure of epidemic of bubonic plague in Europe during medieval times [27, 28]. Other alternative hypotheses attribute this pressure to viral diseases, such as small pox, or viral hemorrhagic fever [27, 28]. But it is still inconclusive. If we ascertain the reasons in the future, it may support an enormous potential approach to obtain sufficient homozygous CCR5 delta 32 cells in vitro to cure AIDS.

The significance of this study is the identification of CCR5 delta 32 heterozygous HEK293T cell line. HEK293T cells could be used as a positive control in genotyping CCR5 allele of other cell lines or individuals in the future. Furthermore, wild type CCR5 and mutant delta 32 DNA can form heteroduplex in PCR and move slowly in gel electrophoresis. It is possible that this heteroduplex double strand DNA could enter into a single bacterium and replicate separately in a single bacterium.

## References

[1] Barre-Sinoussi F, Chermann JC, Rey F, Nugeyre MT, Chamaret S, Gruest J, et al Isolation of a T-lymphotropic retrovirus from a patient at risk for acquired immune deficiency syndrome (AIDS). Science. 1983;220(4599):868–71. . 6189183 10.1126/science.6189183

[2] Wainberg MA, Jeang KT. 25 years of HIV-1 research—progress and perspectives. BMC medicine. 2008;6:31 10.1186/1741-7015-6-31 18976462 PMC2585089

[3] Lopalco L. CCR5: From Natural Resistance to a New Anti-HIV Strategy. Viruses. 2010;2(2):574–600. 10.3390/v2020574 21994649 PMC3185609

[4] Woollard SM, Kanmogne GD. Maraviroc: a review of its use in HIV infection and beyond. Drug design, development and therapy. 2015;9:5447–68. 10.2147/DDDT.S90580 26491256 PMC4598208

[5] Perelson AS, Neumann AU, Markowitz M, Leonard JM, Ho DD. HIV-1 dynamics in vivo: virion clearance rate, infected cell life-span, and viral generation time. Science. 1996;271(5255):1582–6. . 8599114 10.1126/science.271.5255.1582

[6] Lifson JD, Feinberg MB, Reyes GR, Rabin L, Banapour B, Chakrabarti S, et al Induction of CD4-dependent cell fusion by the HTLV-III/LAV envelope glycoprotein. Nature. 1986;323(6090):725–8. 10.1038/323725a0 . 3095663

[7] Lifson JD, Reyes GR, McGrath MS, Stein BS, Engleman EG. AIDS retrovirus induced cytopathology: giant cell formation and involvement of CD4 antigen. Science. 1986;232(4754):1123–7. . 3010463 10.1126/science.3010463

[8] Deng H, Liu R, Ellmeier W, Choe S, Unutmaz D, Burkhart M, et al Identification of a major co-receptor for primary isolates of HIV-1. Nature. 1996;381(6584):661–6. 10.1038/381661a0 . 8649511

[9] Allers K, Schneider T. CCR5Delta32 mutation and HIV infection: basis for curative HIV therapy. Current opinion in virology. 2015;14:24–9. 10.1016/j.coviro.2015.06.007 . 26143158

[10] Heigele A, Joas S, Regensburger K, Kirchhoff F. Increased susceptibility of CD4+ T cells from elderly individuals to HIV-1 infection and apoptosis is associated with reduced CD4 and enhanced CXCR4 and FAS surface expression levels. Retrovirology. 2015;12(1):86 10.1186/s12977-015-0213-1 26452480 PMC4600300

[11] Liu R, Paxton WA, Choe S, Ceradini D, Martin SR, Horuk R, et al Homozygous defect in HIV-1 coreceptor accounts for resistance of some multiply-exposed individuals to HIV-1 infection. Cell. 1996;86(3):367–77. . 8756719 10.1016/s0092-8674(00)80110-5

[12] Walli R, Reinhart B, Luckow B, Lederer E, Loch O, Malo A, et al HIV-1-infected long-term slow progressors heterozygous for delta32-CCR5 show significantly lower plasma viral load than wild-type slow progressors. Journal of acquired immune deficiency syndromes and human retrovirology: official publication of the International Retrovirology Association. 1998;18(3):229–33. . 9665499 10.1097/00042560-199807010-00005

[13] O'Brien TR, Winkler C, Dean M, Nelson JA, Carrington M, Michael NL, et al HIV-1 infection in a man homozygous for CCR5 delta 32. Lancet. 1997;349(9060):1219 . 9130945 10.1016/s0140-6736(97)24017-1

[14] Hutter G, Nowak D, Mossner M, Ganepola S, Mussig A, Allers K, et al Long-term control of HIV by CCR5 Delta32/Delta32 stem-cell transplantation. The New England journal of medicine. 2009;360(7):692–8. 10.1056/NEJMoa0802905 . 19213682

[15] Lewin SR. A cure for HIV: where we've been, and where we're headed. Lancet. 2013;381(9883):2057–8. 10.1016/S0140-6736(13)61180-0 23769215 PMC4062706

[16] Buell KG, Chung C, Chaudhry Z, Puri A, Nawab K, Ravindran RP. Lifelong antiretroviral therapy or HIV cure: The benefits for the individual patient. AIDS care. 2015:1–5. 10.1080/09540121.2015.1074653 . 26357912

[17] Allers K, Hutter G, Hofmann J, Loddenkemper C, Rieger K, Thiel E, et al Evidence for the cure of HIV infection by CCR5Delta32/Delta32 stem cell transplantation. Blood. 2011;117(10):2791–9. 10.1182/blood-2010-09-309591 . 21148083

[18] Wang J, Exline CM, DeClercq JJ, Llewellyn GN, Hayward SB, Li PW, et al Homology-driven genome editing in hematopoietic stem and progenitor cells using ZFN mRNA and AAV6 donors. Nature biotechnology. 2015 10.1038/nbt.3408 . 26551060 PMC4842001

[19] Wang W, Ye C, Liu J, Zhang D, Kimata JT, Zhou P. CCR5 gene disruption via lentiviral vectors expressing Cas9 and single guided RNA renders cells resistant to HIV-1 infection. PloS one. 2014;9(12):e115987 10.1371/journal.pone.0115987 25541967 PMC4277423

[20] Tebas P, Stein D, Tang WW, Frank I, Wang SQ, Lee G, et al Gene editing of CCR5 in autologous CD4 T cells of persons infected with HIV. The New England journal of medicine. 2014;370(10):901–10. 10.1056/NEJMoa1300662 24597865 PMC4084652

[21] Badia R, Riveira-Munoz E, Clotet B, Este JA, Ballana E. Gene editing using a zinc-finger nuclease mimicking the CCR5Delta32 mutation induces resistance to CCR5-using HIV-1. The Journal of antimicrobial chemotherapy. 2014;69(7):1755–9. 10.1093/jac/dku072 . 24651827

[22] Ye L, Wang J, Beyer AI, Teque F, Cradick TJ, Qi Z, et al Seamless modification of wild-type induced pluripotent stem cells to the natural CCR5Delta32 mutation confers resistance to HIV infection. Proceedings of the National Academy of Sciences of the United States of America. 2014;111(26):9591–6. 10.1073/pnas.1407473111 24927590 PMC4084478

[23] Saayman S, Ali SA, Morris KV, Weinberg MS. The therapeutic application of CRISPR/Cas9 technologies for HIV. Expert opinion on biological therapy. 2015;15(6):819–30. 10.1517/14712598.2015.1036736 25865334 PMC4581584

[24] Kang H, Minder P, Park MA, Mesquitta WT, Torbett BE, Slukvin II. CCR5 Disruption in Induced Pluripotent Stem Cells Using CRISPR/Cas9 Provides Selective Resistance of Immune Cells to CCR5-tropic HIV-1 Virus. Molecular therapy Nucleic acids. 2015;4:e268 10.1038/mtna.2015.42 . 26670276

[25] Hu W, Kaminski R, Yang F, Zhang Y, Cosentino L, Li F, et al RNA-directed gene editing specifically eradicates latent and prevents new HIV-1 infection. Proceedings of the National Academy of Sciences of the United States of America. 2014;111(31):11461–6. 10.1073/pnas.1405186111 25049410 PMC4128125

[26] Zhou C, Sun H, Yin JX, Zhang HY, Lin KQ, Tao YF, et al [Detection and preliminary study of a family carrying a CCR5Delta32 deletional mutation]. Zhonghua yi xue yi chuan xue za zhi = Zhonghua yixue yichuanxue zazhi = Chinese journal of medical genetics. 2012;29(4):485–9. 10.3760/cma.j.issn.1003-9406.2012.04.024 . 22875513

[27] Biloglav Z, Zgaga L, Smoljanovic M, Hayward C, Polasek O, Kolcic I, et al Historic, demographic, and genetic evidence for increased population frequencies of CCR5Delta32 mutation in Croatian Island isolates after lethal 15th century epidemics. Croatian medical journal. 2009;50(1):34–42. 19260142 10.3325/cmj.2009.50.34 PMC2657566

[28] Zawicki P, Witas HW. HIV-1 protecting CCR5-Delta32 allele in medieval Poland. Infection, genetics and evolution: journal of molecular epidemiology and evolutionary genetics in infectious diseases. 2008;8(2):146–51. 10.1016/j.meegid.2007.11.003 . 18162443

